# Development and Validation of a Short Version Sport Orientation Questionnaire for Chinese Adolescents (SOQ-CA)

**DOI:** 10.3389/fpsyg.2020.01039

**Published:** 2020-06-17

**Authors:** Jindong Chang, Yali Yi, Naiqing Song

**Affiliations:** ^1^School of Physical Education, Institute of Motor Quotient, Southwest University, Chongqing, China; ^2^School of Mathematics and Statistics, Yulin Normal University, Yulin, China; ^3^School of Mathematics and Statistics, Southwest University, Chongqing, China; ^4^Southwest University Branch, Collaborative Innovation Center of Assessment toward Basic Education Quality, Beijing Normal University, Beijing, China; ^5^Key Liberal Arts Research Bases of Chongqing, Basic Education Research Center, Southwest University, Chongqing, China

**Keywords:** Chinese adolescents, competitiveness, SOQ-CA, sport orientation, sports motivation

## Abstract

A noted decrease in adolescent physical activity in the past decade has resulted in an increase in health risks. Sport orientation correlates closely with physical activity. A sufficient assessment scale that measures an individual’s sport orientation is important to measure an adolescent’s physical inactivity. The purpose of this study was to develop and validate a short version of the Sport Orientation Questionnaire for Chinese Adolescents (SOQ-CA). Based on Gill’s SOQ and previous literature, an initial 30-item questionnaire was developed to create the original SOQ-CA. A five-point Likert scale was used to measure by self-report. In this study, three surveys were conducted. Volunteer participants completed 1,235 valid questionnaires. The data of the first collection sample (*n* = 486) were split randomly into two groups, sample 1 (*n* = 150) used for exploratory factor analysis (EFA) and sample 2 (*n* = 336) for confirmatory factor analysis (CFA). The data of the second (*n* = 377) and third (*n* = 372) collection samples were used to perform test–retest reliability, internal consistency, and CFA of the SOQ-CA. The SOQ-CA obtained good reliability and validity through both EFA and CFA. The development of the SOQ-CA provides an opportunity to develop further theories and practices regarding the assessment of both sport motivation and individual achievement orientation. The application of the SOQ-CA in China would be significant for monitoring the development of adolescent physical activity and aiding in the implementation of policies.

## Introduction

Physical activity is important to adolescent growth and development in any country in the world (Brown et al., 2017). Physical inactivity in youth is a common problem globally, and China is no exception to this (World Health Organization [WHO], 2019). The bulletin of the State General Administration of Sport of China shows that, from 1978 to 2016, physical inactivity in Chinese youth has led to the serious decline of physical fitness (SGASC, 2018). This highlights the fact that we must figure out how to motivate youth participation in physical activity, to improve their physical fitness overall. Some researchers believe that there is a positive link between sport orientation and participation in physical activity (Marsh et al., 1995; Findlay and Coplan, 2008). Sport orientation is an important index that measures individual differences in sport achievement orientation (Gill and Dzewaltowski, 1988; Bowker et al., 2003). It is important to consider the definition of “fit” physical activity types and sport orientation (Kokaridas et al., 2009). One study has noted that “the fit between athleticism and sport orientation are important” (Findlay and Bowker, 2009). Some studies have explored the interaction between sport orientation and an individual’s future competence, confidence, and connectedness (Perkins and Borden, 2004; Telama et al., 2006; Kjønniksen et al., 2009; Miller and Siegel, 2017). Some studies have also suggested that sport orientation may predict competitive anxiety (Jones and Swain, 1992; Daniel et al., 1999). One more recent study showed that fear of competition affects on youth health promotion (Tari-Keresztes et al., 2015). Therefore, revealing the mechanism involved in sport orientation will contribute to the exploration of the reasons for youth physical inactivity (Xu and Gao, 2018).

Previous research has proposed numerous different sport orientations theoretical models—both achievement- and goal-oriented versions—and each model has had a related evaluation tool (Weiner, 1985; Lewthwaite and Piparo, 1993; Mills, 1996; Lata and Mondello, 2010; Gutiérrez et al., 2011; Han et al., 2015). Although the general consensus with these tools is that the measured objective of sport orientation exists, there have been different approaches in how to measure these constructs. For example, the Perceptions of Success Questionnaire (POSQ) and the Task and Ego Orientation in Sport Questionnaire (TEOSQ) have been used to evaluate individual differences in task and ego goal proneness (Duda, 1989; Roberts and Balagué, 1989; Lochbaum et al., 2016). The Competitive Orientation Inventory (COI) was developed to measure an athlete’s self-confidence and competitiveness orientation (Vealey, 1986). The Sport Orientation Questionnaire (SOQ) and the Achievement Motives Scale-Sport (AMS-Sport; German version) were both designed to assess sport-specific achievement (motivation) orientations (Gill and Deeter, 1988; Elbe, unpublished). Today, the Gill’s SOQ has been used extensively to assess sport-specific orientation (Gill et al., 1996; Skordilis and Stavrou, 2005; Skordilis et al., 2006; Shihui et al., 2007; Swann et al., 2015). Although the measurement constructs of the Gill’s SOQ and the other tools differ, all five questionnaires evaluate the same achievement goal orientation (Duda and Whitehead, 1998; Hanrahan and Biddle, 2002). As the Gill’s SOQ has been shown to effectively measure competitiveness and achievement behavior in sport and exercise settings, the present study chose to revise the SOQ as a measurement tool of sport orientation for Chinese adolescents (Lihui, 2004).

Gill’s SOQ is “a multidimensional, sport-specific measure of individual differences in sport achievement orientation” (Gill and Deeter, 1988). It is a 25-item scale that includes three subscales: competitiveness orientation, win orientation, and goal orientation (Gill and Deeter, 1988). The competitiveness orientation subscale consists of 13 items that measure participants’ feelings regarding their competition with others (desire for competition). The win orientation subscale comprises six items that measure participants’ perceptions regarding success in competition (focus on winning). The goal subscale is made of six items that assess participants’ feelings regarding their sports performance (personal performance). The scale is rated on a five-point Likert scale (1 = strongly disagree, 5 = strongly agree) and the level of internal consistency is high, with a Cronbach’s α of 0.88.

Gill’s SOQ has been used widely since its development to assess and predict sport achievement orientation. Trembath et al. (2002), Biddle et al. (2003), Kolt et al. (2004), Daniels et al. (2005), Sheikh et al. (2011), Farshad et al. (2013), and Baron-Thiene and Alfermann (2015) have all reported appropriate models of the scale and confirmed that the SOQ was effective at measuring certain athletic tendencies among numerous sample communities. Studies have shown that there are significant differences between the goal task and participation motivation among different sports levels of athletes, such as amateur athletes and professional athletes (Koumpoula et al., 2011; Alfermann et al., 2013). Sports motivation, sports participation, and sports competitiveness have been used and have been shown to be effective models for evaluating certain athletes (e.g., elite athletes or disabled athletes) (Zarghmi et al., 2010; Scoffier et al., 2012; Allen et al., 2015). However, there are only a few scholars who have focused specifically on adolescent sport orientation (Vilkas and Mëlinis, 2017; Chang et al., 2018).

There is already a large amount of published literature that has examined sport orientation and sport motivation that focuses on goal orientation or achievement motivation constructs (Dweck, 1986; Maehr and Braskamp, 1986; Vealey, 1986; Stipek, 1988; Nicholls, 1989; Ames, 1992; Roberts, 1992; Duda, 1993; Treasure and Roberts, 1994). Past research has used both exploratory (Duda, 1989; Duda and White, 1992; Newton and Duda, 1993; Kim and Gill, 1997; Skordilis et al., 2001; Wakayama et al., 2002; Tari-Keresztes et al., 2015) and confirmatory (Kang et al., 1990; Li and Tang, 1990; Seifriz et al., 1992; Hayashi and Weiss, 1994; Marsh, 1994; Chi and Duda, 1995; Duda, 1995; Lerner and Locke, 1995; Skordilis et al., 2001; Hanrahan and Biddle, 2002) factor analysis to test the construct of the Gill’s SOQ. However, sample participants have been largely sourced from the United States, Australia, or the European Union. Very few studies have reported results from Asian or Middle Eastern contexts, though there has been published research from Iran (Farshad et al., 2013), Japan (Wakayama et al., 2002), and China (Kang et al., 1990; Chang et al., 2018).

Gill’s SOQ has already been adapted to create a Chinese version of the measurement, revised and validated using college students and elite athletes (Li and Tang, 1990). However, a recent study has reported that Gill’s SOQ (Chinese version) cannot adequately distinguished Chinese youth sport orientation, particularly in differentiating between competitiveness and win orientations (Chang et al., 2018). Additionally, an earlier study found that Gill’s SOQ (Japanese version) differed when compared back to the original English-language version in terms of competitiveness (Wakayama et al., 2002). One study in Taiwan found sex differences only in competitiveness (Kang et al., 1990), while another study on Koreans indicated that there was no significant gender difference in the sport orientation (Kim and Gill, 1997). The differences in sample demographics and cultures may lead to differences in the results and their meanings when comparing translated versions of the SOQ to the original English version (White et al., 1998). Consequently, in revising the Chinese version of the SOQ, it is important that study sample demographics, and cultural differences must be considered first (Bernstein and Nunnally, 1994). The purpose of the present study then was to develop and validate the Chinese version of the SOQ by relating responses to items adapted from Gill’s original SOQ (1988) as well as to external criteria, specifically to multiple dimensions of subjects’ physical self-concepts.

## Materials and Methods

### Participants

The participants consisted of two groups. The first group was part of a 30-item SOQ exploration phase, made up of a total of 486 individuals (male = 249, female = 237). Their age ranged from 12 to 16 years (13.63 ± 1.38). The participants of group 1 were split randomly using a computer-generated randomization sequence (GraphPad Software, Inc.) into two subgroups with the approximate ratio of 1:2 (i.e., sample 1 = 150 cases, sample 2 = 336 cases) (Sum et al., 2016), in which the subsample size satisfied the minimum amount of data required (Paiva et al., 2014). Participants in group 2 performed two tests. The subgroup that completed first test was named sample 3 (*n* = 377), and the subgroup that completed the second test was sample 4 (*n* = 372). Group 2 participants completed the 13-item SOQ-CA, and comprised 338 participants (male = 180, female = 158, age = 13.18 ± 0.86). Considering that the academic performance of Chinese urban school students is better than that of rural schools, the universality of the questionnaire was verified by using samples from two different regional schools. The participants in group 1 came from two middle schools in central Chongqing, China. The participants in group 2 came from three middle schools in the urban–rural junction in Chongqing, China (Table 1).

**TABLE 1:** Basic characteristics of participants.

	Group 1	Sample 1	Sample 2	Sample 3/4
Sex (Male/Female)	249/237	69/81	180/156	180/158
Age (Years)	12–16	12–16	12–16	12–16
BMI	19.8 ± 3.65	19.8 ± 3.87	19.8 ± 3.57	19.7 ± 4.16
Sports level (active)	47	17	30	47
Sports level (general)	439	133	306	291

### Procedures

All participants voluntarily agreed to take part in the study. As they were all minors, their guardians approved their participation in the study by signing the research information letter. The questionnaire was distributed and collected with the assistance of the class teacher and was completed in the classroom before school extracurricular activities. The researchers were responsible for answering any questions raised by the participants. The average questionnaire completion time was 6 min. The data for group 1 (samples 1 and 2) were collected between March and June of 2018, and all these participants agreed to complete the 30-item SOQ and AMS-Sport questionnaires. Although the data collection lasted 2 months, samples 1 and 2 were not involved in a time interval because these data were split from group 1. The data for samples 3 and 4 were collected during May and June of 2019, with these data collected at 15-day intervals. These participants completed the 13-item Chinese version SOQ.

### Measures

#### Scale Development

First, Gill’s SOQ English version was translated to create the SOQ Chinese version. Based on the literature review and interviews with sports motivation research experts, 14 items were added, making the SOQ Chinese version a 39-item questionnaire. Once this was developed, it was submitted to three professional researchers for conceptual evaluation: one researcher with experience in sports exercise and adolescent sports motivation; one with experience in adolescent sports motivation and the development and validation of instruments; and one with experience in sports competitiveness, adolescent health, and goal orientation. The SOQ Chinese version was evaluated by all three researchers from the following perspectives: semantic equivalence, conceptual scope, clarity, readability, relevance, and conciseness (Patton, 1999; Yin, 2002; Paiva et al., 2014). During this process, seven items were deleted because of low relevance, and four items were merged into two items due to lack of clarity. The final questionnaire, then, including 30 items (see Table 2). After all three researchers agreed that none of the items required further modification, the SOQ Chinese version was sent to a linguistics professor to check its grammar.

**TABLE 2:**
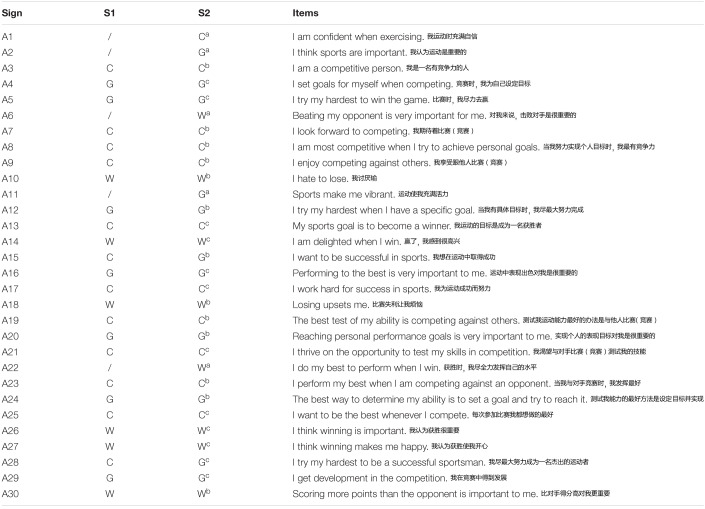
SOQ Chinese version items containing 30 questions on sport orientation.

*All questions are rated on a five-point Likert scale (1 = strongly disagree, 5 = strongly agree). C, competitiveness orientation; G, goal orientation; W, win orientation; S1, factor distribution of Gill’s SOQ; S2, factor distribution of new SOQ Chinese version. ^a^The new statement. ^b^The original statement. ^c^The changed statement.*

Second, an interview was conducted using a focus group composed of a sample of 12 adolescents (group 1 with two schools, group 2 with one school, and each school with four students) to test their understanding of the questionnaire items (Boynton and Greenhalgh, 2004). All participants were chosen randomly, following the criteria of having regular enrollment in school, being 12–16 years of age, having received authorization from their parents through a signed statement of informed consent, and having received the student’s agreement to participate. Focus group participants shared their views on sport orientation and discussed the relationship between sports goals, sports motivation, sports competitiveness, and sport orientation. The researchers used structured questions to guide the focus group discussion, which helped to identify critical points such as competitiveness, orientation, goal, and motivation to aid in the instrument’s development. After the focus group, the questionnaire was revised using the suggestions proposed by the interviewees. At this point, the new version of the instrument was sent to two external researchers for further review.

Third, a 30-item preliminary Chinese version of SOQ was developed (see Table 2). The 30-item version included the translated items from Gill’s SOQ—of which 13 items that were left unchanged (items 3, 7, 8, 9, 10, 12, 15, 18, 19, 20, 23, 24, and 30), and 12 items that were revised (items 4, 5, 13, 14, 16, 17, 21, 25, 26, 27, 28, and 29)—and included five additional items (items 1, 2, 6, 11, and 22). With regard to the revised items, some clarifications were added to items because of linguistic details of the Chinese language (i.e., “I think” was added to item 25), some changes were to add objects (i.e., “the game” was inserted into item 5), and some questions underwent major changes (i.e., item 27 changed “I have the most fun when I win” to “I think winning makes me happy”). The five new items were designed to effectively distinguish between goal orientation (items 2 and 11) and win orientation (items 6 and 22), and one final item to examine competitive factors (item 1).

#### Reliability

Test–retest and internal consistency were used in this study to verify the reliability of the SOQ-SA (Singh et al., 2011; Mehta et al., 2018). In general, α coefficients above 0.70 indicate acceptable (Barker et al., 1994). In evaluating the internal consistency, items with an item-to-total correlation of less than 0.20 were removed (McGraw and Wong, 1996).

#### Validity

The content validity of the questionnaire was performed by nine psychology professors with experience in sport motivation and sport orientation studies (Paiva et al., 2014). Professors were asked to review each item in the questionnaire and evaluate their relevance to the measured objects, clarity, and conciseness of each item.

Exploratory factor analysis (EFA) was applied to evaluate the questionnaire’s construct validity. Bartlett’s test and the Kaiser–Meyer–Olkin (KMO) measure were performed to test whether the data were appropriate for factor analysis. KMO values above 0.7 indicated that the condition for factor analysis was met (Bolarinwa, 2015). Generally, for a sample of 150 individuals, factor loadings greater than 0.50 are considered significant (Paiva et al., 2014). Factors were extracted considering eigenvalues greater than one and orthogonal rotation was performed using the varimax and direct oblimin method (Esbensen et al., 2002). If similar items displayed the load under different factors, the items would be deleted (Auxter et al., 2005).

The criterion-related validity between achievement orientations (AMS-Sport) and the SOQ-CA was analyzed based on sample 1 and sample 2. The AMS-Sport consists of two components: hope for success (HS) and fear of failure (FF). Each component has 15 items. The sum of the scores for each item is the total achievement motivation. Cronbach’s α of the AMS-Sport was 0.90, and the measure’s test–retest reliability was 0.71 (Elbe and Wenhold, 2005).

### Data Analysis

Researchers analyzed any missing values in returned questionnaires. Frequency statistics showed that there were 36 cases in which some values were missing. Researchers used the mean to estimate missing values, allowing for all survey data collected to be included in the analysis (Field, 2009).

Exploratory factor analysis was performed on the data from sample 1, using IBM SPSS 24.0. Confirmatory factor analysis (CFA) was performed on the data from samples 2, 3, and 4, using AMOS 24.0 (SPSS Inc., Chicago, IL, United States). In the CFA verification, values for comparative fit index (CFI), goodness of fit index (GFI), Normed fit index (NFI), and Tucker-Lewis index (TLI) were all greater than 0.90, which meant that the model was considered to be a good fit (Pons et al., 2006). For the root mean square error of approximation (RMSEA), a value of 0.06 suggests a good fit, and 0.08 is an acceptable fit (Bentler and Bonett, 1980).

## Results

### Descriptive Statistics

Table 3 shows the means and standard deviations of the SOQ-CA for all four samples.

**TABLE 3:** Means and standard deviations of SOQ-CA.

Item	Sample 1 (*n* = 150)		Sample 2 (*n* = 336)		Sample 3 (*n* = 338)		Sample 4 (*n* = 338)	
	*M*	*SD*	*M*	*SD*	*M*	*SD*	*M*	*SD*
A2	4.33	1.14	4.28	1.19	4.37	0.93	4.46	0.90
A4	3.87	1.29	3.77	1.33	3.91	1.15	3.89	1.16
A5	4.39	1.07	4.12	1.26	4.28	1.00	4.35	0.99
A6	3.26	1.33	3.10	1.44	3.28	1.33	3.24	1.33
A7	3.33	1.40	3.13	1.47	3.31	1.36	3.28	1.36
A8	3.52	1.32	3.30	1.33	3.49	1.19	3.51	1.19
A9	3.47	1.33	3.40	1.43	3.55	1.25	3.53	1.31
A10	3.34	1.46	3.16	1.47	3.30	1.39	3.33	1.41
A11	3.75	1.45	3.79	1.33	3.87	1.20	3.92	1.16
A14	2.43	1.44	2.25	1.41	2.41	1.39	2.45	1.35
A18	2.99	1.42	2.94	1.44	3.07	1.33	3.10	1.31
A26	3.20	1.44	3.10	1.47	3.24	1.39	3.26	1.36
A30	2.71	1.44	2.56	1.39	2.78	1.35	2.67	1.31
Total	44.59	17.52	42.91	17.96	44.86	16.27	44.99	16.14

### Exploratory Factor Analysis

The initial questionnaire consisted of 30 items. The data from the first sample (*n* = 150) on the 30 items were evaluated by EFA (see Table 1). Seventeen items were removed after EFA, and 13 items were retained (see Table 4). The 13-item questionnaire demonstrated item-total correlations ranging from 0.59 to 0.71; the KMO index was 0.86, Bartlett’s test of sphericity proved to be significant (*p* < 0.001), and Cronbach’s α coefficient for the total scale was 0.88 (see Table 4).

**TABLE 4:** Factor structures by exploratory factor analysis and reliability (*n* = 150).

Sign	Items	Win		Goal		Competitiveness		Var TVE%	ITC-S	ITC-F	α
		Var	Olb	Var	Olb	Var	Olb				
A6	Beating my opponent is very important for me.	**0.72**	0.71	0.15	0.00	0.28	0.21	27.53	0.77	0.71	0.87
A10	I hate to lose.	**0.77**	**0.80**	0.09	0.00	0.02	−0.08		0.76	0.59	
A14	I am delighted when I win.	**0.80**	**0.83**	−0.00	−0.15	0.16	0.10		0.80	0.63	
A18	Losing upsets me.	**0.76**	**0.77**	0.07	−0.07	0.21	0.13		0.78	0.66	
A26	I think winning is important.	**0.71**	**0.69**	0.34	0.29	0.01	−0.15		0.76	0.67	
A30	Scoring more points than the opponent is important to me.	**0.76**	**0.77**	0.17	0.09	0.03	−0.09		0.78	0.63	
A2	I think sports are important.	0.16	0.04	**0.81**	**0.86**	0.12	−0.09	21.09	0.81	0.61	0.83
A4	I set goals for myself when competing.	0.07	−0.08	**0.80**	**0.81**	0.32	0.16		0.86	0.64	
A5	I try my hardest to win the game.	0.24	0.12	**0.72**	**0.70**	0.28	0.11		0.80	0.68	
A11	Sports make me vibrant.	0.10	−0.03	**0.72**	**0.72**	0.29	0.13		0.82	0.61	
A7	I am looking forward to competing.	0.19	0.08	0.26	0.06	**0.79**	**0.80**	17.54	0.86	0.65	0.81
A8	I am most competitive when I try to achieve personal goals.	0.17	0.06	0.28	0.10	**0.73**	**0.73**		0.82	0.62	
A9	I enjoy competing against others.	0.08	−0.04	0.27	0.07	**0.83**	**0.85**	66.16	0.87	0.61	0.88

*α, Cronbach’s α; ITC-F, Item-Total Correlation (full scale); ITC-S, Item-Total Correlation (subscales); TVE%, Total Variance Explained. Bold numbers represent significant correlation in this dimension (p < 0.001).*

Exploratory factor analysis of the 13-item questionnaire produced three factors: win orientation, goal orientation, and competitiveness orientation. The win orientation factor included six variables (i.e., items 6, 10, 14, 18, 26, and 30), the goal orientation factor included four variables (i.e., items 2, 4, 5, and 11), and the competitiveness orientation factor included three variables (i.e., items 7, 8, and 9). This means EFA resulted in the SOQ-CA containing 12 fewer items than Gill’s SOQ (1988). Decreasing item numbers boosted the measurement’s efficiency, but the reliability of the questionnaire required further verification (Petróczi, 2007; Manouchehri and Tojari, 2013). The total variance explained of each factor is shown on the diagonal in Table 4. These three factors explained 66.16% of the variance in the data. The win orientation alone explained 27.53% of the overall variability in the data; goal orientation explained 21.09% of the overall variability, and competitiveness orientation explained17.54% of the overall variability (see Table 4). The correlations among the three facets of the four samples are also shown in Table 5. The pattern shown is generally consistent between both samples. Age is positive relation with the SOQ-CA (*p* < 0.01); group is negative relation (*p* < 0.01). However, sex is shown no related to the SOQ-CA in all four samples.

**TABLE 5:** Correlations among variables in samples 1, 2, 3, and 4.

Sample 1 (*n* = 150)	Sex	Age	BMI	Group	Win	Goal	Competitiveness
Win	0.045	0.512^∗∗^	0.022	−0.362^∗∗^			
Goal	0.051	0.433^∗∗^	0.127	−0.198^∗∗^	0.370^∗∗^		
Competitiveness	–0.119	0.404^∗∗^	–0.018	−0.371^∗∗^	0.360^∗∗^	0.604^∗∗^	
SOQ	0.008	0.584^∗∗^	0.053	−0.400^∗∗^	0.836^∗∗^	0.768^∗∗^	0.739^∗∗^
**Sample 2 (*n* = 336)**							
Win	–0.075	0.351^∗∗^	–0.022	−0.306^∗∗^			
Goal	–0.057	0.328^∗∗^	–0.059	−0.148^∗∗^	0.415^∗∗^		
Competitiveness	–0.116	0.416^∗∗^	–0.015	−0.295^∗∗^	0.446^∗∗^	0.722^∗∗^	
SOQ	–0.097	0.438^∗∗^	–0.038	−0.312^∗∗^	0.841^∗∗^	0.806^∗∗^	0.867^∗∗^
**Sample 3 (*n* = 338)**							
Win	0.022	0.228^∗∗^	0.050	−0.337^∗∗^			
Goal	–0.037	0.081	0.049	−0.203^∗∗^	0.196^∗∗^		
Competitiveness	–0.025	0.167^∗∗^	–0.010	−0.206^∗∗^	0.305^∗∗^	0.537^∗∗^	
SOQ	–0.006	0.233^∗∗^	0.046	−0.359^∗∗^	0.827^∗∗^	0.645^∗∗^	0.713^∗∗^
**Sample 4 (*n* = 338)**							
Win	0.030	0.223^∗∗^	0.032	−0.361^∗∗^			
Goal	–0.007	0.084	0.100	−0.211^∗∗^	0.238^∗∗^		
Competitiveness	0.022	0.130^∗^	0.028	−0.230^∗∗^	0.291^∗∗^	0.565^∗∗^	
SOQ	0.024	0.215^∗∗^	0.064	−0.381^∗∗^	0.821^∗∗^	0.685^∗∗^	0.714^∗∗^

*^∗^ p < 0.05, ^∗∗^ p < 0.01. The average variance extracted of three factors are shown on the diagonal. Group included active group and general group, 1 = active, 2 = general.*

Meanwhile, to assess the fit of factor models, we examined the differences between the model-based correlations and the observed correlations three-factor model, which showed that 69% of the residuals were less than 0.1 (Cronbach, 1951). Regarding the internal consistency of three factors, all factors showed that Cronbach’s alpha above 0.70 was sufficiently good (see Table 4).

### Confirmatory Factor Analysis

**FIGURE 1:**
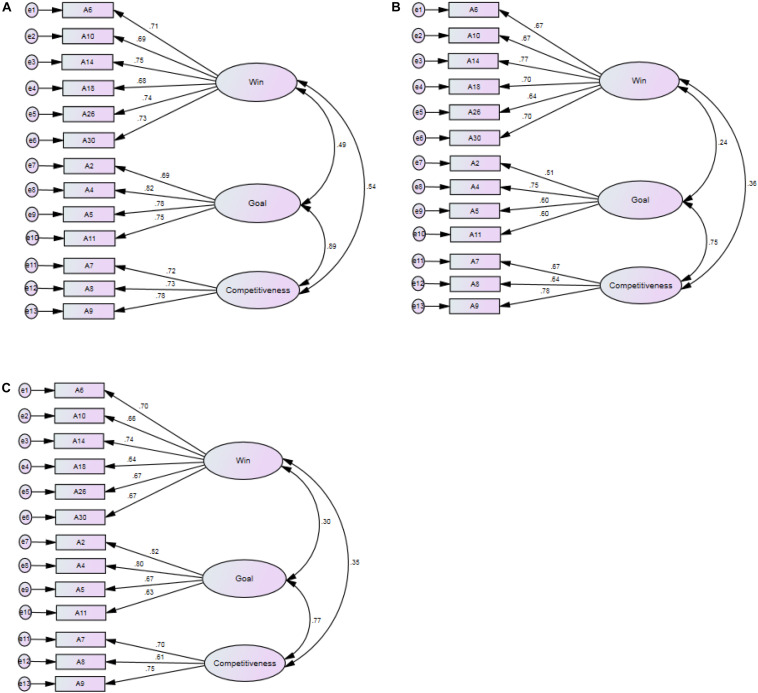
Factor structure and standardized factor loading on sport orientation items. **(A)** Factor structure and standardized item loading for SOQ of Sample 2. **(B)** Factor structure and standardized item loading for SOQ of Sample 3. **(C)** Factor structure and standardized item loading for SOQ of Sample 4.

The data from sample 2 (*n* = 336), sample 3 (*n* = 338), and sample 4 (*n* = 338) were used to validate and confirm the three-factor structure produced by the previous EFA. The results of the CFA indicated that the three-factor model had an adjusted model fit indices: sample 2 (χ^2^/*df* = 150.458/62 = 2.427, *p* < 0.001), CFI = 0.96, GFI = 0.94, NFI = 0.93, TLI = 0.95, RMSEA = 0.065; sample 3 (χ^2^/*df* = 153.978/62 = 2.484, *p* < 0.001), CFI = 0.93, GFI = 0.94, NFI = 0.89, TLI = 0.92, RMSEA = 0.066; sample 4 (χ^2^/*df* = 143.149/62 = 2.309, *p* < 0.001), CFI = 0.94, GFI = 0.94, NFI = 0.90, TLI = 0.93, RMSEA = 0.062. The indicators showed that all the model fitting indices met the standard. Furthermore, the item loadings ranged from 0.52 to 0.82 (see Figures 1A–C) and all were above the standard of 0.45 (see Figure 1). The factor loading of all items was significant (*p* < 0.05). These results indicated that the three-factor scale structure was satisfactory (Horn and McArdle, 1992; Hu and Bentler, 1999).

### Reliability of the SOQ-CA

The data from samples 2, 3, and 4 were examined to test the internal consistency of the SOQ-CA (see Table 6). The results showed that win orientation (0.86, 0.87, and 0.86, respectively), goal orientation (0.85, 0.86, and 0.80, respectively), and the SOQ-CA overall (0.89, 0.90, and 0.87, respectively) scored above 0.80. The competitiveness orientation was close to 0.80 in sample 2 (0.79) and sample 4 (0.78).

**TABLE 6:** Internal consistency and test–retest reliability for the SOQ-CA.

	Cronbach’s α Sample 2 (*n* = 336)	Cronbach’s α Sample 3 (*n* = 338)	Cronbach’s α Sample 4 (*n* = 338)	Test–retest reliability Samples 3 and 4 (*n* = 338)
Win	0.86	0.87	0.86	0.81
Goal	0.85	0.86	0.80	0.76
Competitiveness	0.79	0.80	0.78	0.76
SOQ-CA	0.89	0.90	0.87	0.94

The data from sample 3 and 4 were examined to validate the test–retest reliability of the SOQ-CA. The test–retest reliability was 0.81 for the win orientation, 0.76 for the goal orientation, 0.76 for the competitiveness orientation, and 0.94 for the SOQ-CA overall (see Table 6). All test–retest reliabilities scored above 0.75, indicating that the SOQ-CA had higher stability (McGraw and Wong, 1996).

### Validity of the SOQ-CA

To confirm the validity of the construct, correlations between the SOQ-CA and AMS-Sport were analyzed using data from samples 1 and 2. The results are presented in Table 7. The components of the SOQ-CA were correlated with the scales of the AMS-Sport. In both sample 1 and sample 2, the HS scales of the AMS-Sport correlated significantly with those of win orientation (*r* = 0.35; *r* = 0.37, respectively), goal orientation (*r* = 0.53; *r* = 0.54, respectively), and competitiveness orientation (*r* = 0.51; *r* = 0.53) of the SOQ-CA. Goal orientation (*r* = 0.17; *r* = 0.16, respectively) and competitiveness orientation (*r* = 0.18 for sample 2) also correlated with the FF scale of the AMS-Sport. There was no significant correlation between the win orientation and the FF in either sample 1 or sample 2 (see Table 7).

**TABLE 7:** Correlations between the scales of the SOQ-CA and AMS-Sport.

	Sample 1 (*n* = 150)		Sample 2 (*n* = 336)	
	HS	FF	HS	FF
Win orientation	0.35**	0.10	0.37**	0.11
Goal orientation	0.53**	0.17**	0.54**	0.16**
Competitiveness	0.51**	0.16**	0.53**	0.18**

** p < 0.01.

To further verify the construction of validity, different levels of physical activity were additionally examined (Litwin, 1995). In the context of the questionnaire, physical activity frequency was surveyed. The frequency of physical activity for 1 h or more and more than five times per week was defined as the active group; physical activity less than five times a week, or less than 1 h each time was defined as the general group. Univariate analysis of variance was performed on the data from samples 1, 2, 3, and 4. The ANOVA results show that the active group score was higher than that of the general group, with a significant difference visible in all samples (Table 8).

**TABLE 8:** ANOVA among variables in samples 1, 2, 3, and 4.

Samples	Group	Win	Goal	Competitiveness	SOQ
1	Active group (*n* = 17)	24.59 ± 5.24	18.59 ± 2.40	13.88 ± 1.32	57.06 ± 5.76
	General group (*n* = 133)	17.08 ± 6.28	16.05 ± 4.15	9.87 ± 3.36	43.00 ± 10.71
	*F*	22.28	6.06	23.59	28.14
	*P*	0.000***	0.015*	0.000***	0.000***
2	Active group (*n* = 30)	23.60 ± 6.69	17.97 ± 3.85	13.17 ± 2.88	54.73 ± 12.40
	General group (*n* = 306)	16.48 ± 6.3	15.77 ± 4.23	9.50 ± 3.44	41.75 ± 11.18
	*F*	34.48	7.48	31.89	36.11
	*P*	0.000***	0.007**	0.000***	0.000***
3	Active group (*n* = 42)	23.57 ± 5.36	18.12 ± 2.27	12.02 ± 3.05	53.71 ± 7.97
	General group (*n* = 296)	17.30 ± 5.85	16.19 ± 3.18	10.11 ± 3.01	43.60 ± 8.8
	*F*	43.02	14.38	14.87	49.71
	*P*	0.000***	0.000***	0.000***	0.000***
4	Active group (*n* = 42)	23.79 ± 5.77	18.40 ± 2.41	12.21 ± 3.08	54.40 ± 8.95
	General group (*n* = 296)	17.25 ± 5.57	16.36 ± 3.22	10.04 ± 3.03	43.65 ± 8.60
	*F*	50.23	15.68	18.83	56.99
	*P*	0.000***	0.000***	0.000***	0.000***

* p < 0.05, ** p < 0.01, *** p < 0.001.

## Discussion

Based on the theoretical frameworks of Gill’s SOQ, this study developed and validated a novel instrument to measure adolescent sport orientation in a Chinese context. The resulting SOQ-CA is composed of three scales (13 items total): win orientation (six items), goal orientation (four items), and competitiveness orientation (three items). Win orientation (i.e., items 6, 10, 14, 18, 26, and 30) assessed one’s willingness to win. Goal orientation (i.e., items 2, 4, 5, and 11) assessed one’s sport goal-setting by winning a game or sporting experience. Competitiveness (i.e., items 7, 8, and 9) measured one’s sense of competition by looking forward to the game, enjoying the game, and achieving the goal of the game.

In addition, the SOQ-CA shows satisfying test-control criteria. The internal consistency of the SOQ-CA scored above 0.80 and the retest reliability above 0.75, indicating that the SOQ-CA has satisfactory validity and reliability (Barrow and McGoe, 1964; McGraw and Wong, 1996). The confirmatory analysis of the three samples also achieved satisfactory results (Tabachnick and Fidell, 2007). Further proof of the validity of the SOQ-CA construction is that the score of the active group is significantly higher than that of the general group. It indicates that the three-factor model is sufficient to measure the sport orientation in adolescents with different activity levels (Petróczi, 2003). However, the results of the CFA showed that the goal orientation and competitiveness factors were highly correlated (Rose et al., 1998). The reasons for this high correlation require further analysis.

Although the naming of the SOQ-CA subscales is consistent with Gill’s SOQ, the measurement objective and goal of SOQ-CA are different from those of Gill’s SOQ. Specifically, the goal of Gill’s SOQ is to distinguish the athletic tendencies of elite athletes, while SOQ-CA focuses on youth. Gill’s SOQ is more inclined to tap into the competitive potential of athletes, while the SOQ-CA tends to tap into the potential for student activity. It is worth mentioning that some previous studies have indicated that there are significant gender differences in sport orientation among elite athlete (Gill and Deeter, 1988; Findlay and Bowker, 2009; Jamshidi et al., 2011).

Another study has reported that there is no gender difference noted in either the competitiveness orientation (Martin et al., 1995) or the win orientation (Martin and Gill, 1995). This indicates controversy in regard to the issue of gender differences within sport orientation. However, this current study was not unable to address this issue.

In the construction of SOQ-CA, Chinese adolescents are more interested in factors winning (items 6, 14, and 26) than scoring (item 30). This seems to indicate that the act of winning itself is more important than winning more points. However, in interpreting these data to aid a particular adolescent, it is essential to consider whether winning is an important part of the sport (Manouchehri et al., 2013). Winning must be kept in perspective alongside other valuable aspects of youth sport, such as social development, fun, and fitness (David, 2019). Winning a competition is not a prerequisite for future life success, and it is more important to promote all three orientations, not merely winning, in whatever activity adolescents choose to do (Wigfield and Cambria, 2010). At the same time, as one of only three subscales in the SOQ-CA, the win orientation is also a necessary aspect of sport orientation in adolescents. This is consistent with Franken’s view that the need to win tends to correlate with one’s ego orientation (Franken and Brown, 1996). Malhotra (2010) argues that elements of the strategic environment can fuel competitive motivations and behaviors. The desire to win is further heightened when rivalry and time pressure coincide. Therefore, from an applied perspective, teachers and parents may find it beneficial to target individual interventions designed to enhance win ability (Jeffrey and Diane, 1995).

Competitiveness orientation is defined as a desire to enter and strive for success in sports competition (Martens, 1976; Vandewalle, 1997; Gill and Deeter, 1988; Kohn, 1992; Shields and Bredemeier, 2011). The concept of goal orientation, meanwhile, defines one’s disposition toward developing or validating one’s ability in achievement settings (Roberts and Ommundsen, 1996; Vandewalle, 1997; White et al., 1998). Individual goals serve as organizing principles, influencing the meaning of activities and how individuals respond to success (White and Zellner, 1996). In competitive sports situations, competitiveness orientation is influenced by individual differences and situational factors (Gill and Deeter, 1988; Martin and Gill, 1991). Meanwhile, the dimensions of goal orientation are constructed by recognizing the value of sports, goal-setting, working hard to win, and vibrancy in the activity. Compared to competitiveness orientation, goal orientation seems to be less affected by specific sport situations (Gill and Deeter, 1988). However, the SOQ-CA shows that competitiveness and goal orientations are highly correlated, which leads us to question the influence of both goal and competitiveness orientation (Newby and Klein, 2014; Clancy et al., 2016). Monacis et al. (2013) indicates that there is, in fact, a mediating effect between goal and competition orientations. Tari-Keresztes et al. (2015) state that sport situations cannot be ignored as a dimension of the construct of competitiveness orientation. In Gill’s SOQ, competitiveness and goal orientations reflect ego orientation and task orientation as two separate motivators (Gill, 1993). However, competitiveness may become intrinsically connected to one’s task orientation when it relates to internal goals or standards (Monacis et al., 2013). Consequently, one’s task orientation in these circumstances may measure higher (Jensen, 1999; Jones et al., 2006; Wright et al., 2016). Future research should investigate the specific mechanism at play between goal orientation and competitiveness, especially focusing on the mediating role of sports situations.

Finally, one limitation of this study is the fact that our sample participants all came from the same geographic location, which means that our results cannot be assumed to represent all adolescents on a national scale without further verification. Additionally, this study limited participant age to 12–16 years. Future research should expand to include nationwide samples, a wider variety of ages, and different sport types (i.e., football, tennis, and jogging) to improve the questionnaire’s reliability and validity better.

## Conclusion

A novel 13-item short Chinese version of Sport Orientation Questionnaire (SOQ-CA) was developed based on the frameworks of Gill’s original SOQ. With the analysis of data from Chinese youth, it has been proven to be a reliable and valid measure of sport orientation for Chinese adolescents. It can be used not only to assess daily exercise or physical education in class, but also for adolescent self-evaluation to assess daily exercise or physical education in class, as well as adolescent’s self-evaluation regarding their extracurricular physical activities and competitive orientation.

## Data Availability Statement

The raw data supporting the conclusions of this article will be made available by the authors, without undue reservation, to any qualified researcher.

## Ethics Statement

This study was approved by the Scientific Ethics Committee of Academic Committee of the Southwest University. All parents/guardians signed a statement of consent authorizing the participation of their children. All the adolescents also signed the consent forms.

## Author Contributions

JC and YY: data collection, conception and design, data analysis, and writing the manuscript. NS: research design and critical revision.

## Conflict of Interest

The authors declare that the research was conducted in the absence of any commercial or financial relationships that could be construed as a potential conflict of interest.
